# The Medical Expert—What He Is and What He Should Be

**Published:** 1897-10

**Authors:** Hugh Hagan

**Affiliations:** Atlanta


### CORRESPONDENCE.

### THE MEDICAL EXPERT—WHAT HE IS AND WHAT HE SHOULD BE.

ATLANTA, September 10, 1897.

*Atlanta Medical and Surgical Journal:*

Of the many branches of a progressive artistic science, if I may use the expression, not one branch is of more importance to the municipal government, or body politic, than that known as forensic medicine, or medico-legal jurisprudence.

In strong contrast to the usual sacred secrecy of medical cases stands the publicity necessarily attending forensic cases.

The world with one accord applauds the medical adviser, who is wise and considerate enough to let his patient's condition be the patient's secret; and likewise with one accord does the same world condemn the medical attendant who wilfully or thoughtlessly gives the patient's secret to the world.

In striking comparison or contrast stands the medical expert, who is expected to tell all, and maybe more than he knows.

Under the code of medical ethics and the law of medico-legal jurisprudence, he must tell the results of his examination, and his ideas in regard to the case in question, or possibly a supposititious case.

When he, the medical witness, is summoned by the court to appear before it, he is often in ignorance of the case, and likewise as to which side had him called; but again he will know and possibly have stipulated, the amount of the honorarium. In the first case it were easy for him to unbiasedly examine and testify; where it were psychologically difficult in the second, even though nothing more be expected of him than the benefit of the doubt.

We are all cognizant of the psychological fact, that to achieve absolute judicial equanimity and equipoise is only to be acquired after much severe schooling; ergo, having been summoned, it is presupposed he is qualified to render such professional service as the layman cannot. He is supposed to say, he knows or does not know; that he thinks or does not think, or believes or does not believe. No circumlocution or hemming or hawing; likewise no dogmatic statements; for, though having the courage of one's convictions, one must remember the liability of things human to err.

He is in the position of interpreter, and is expected to so express himself that the jury will not be "in confusion worse confounded." He should above all things have the courage to acknowledge conscious knowledge, or conscious ignorance.

Not denying the much deplored fact that men equally honest, intelligent, educated and experienced do differ in their opinions and judgments, it should be possible to obtain a diagnosis sufficiently positive on which to base a correct and sensible verdict. If the expert witness, I say expert unadvisedly, were able to conceive, comprehend and acknowledge that no man knows all medicine, but that some know more than others, intelligent verdicts would be the rule and not the exception.

When our Alma Mater gave us her blessing and diploma, she only said well done, not perfect, for well she knew time, effort and experience were necessary for even the greatest intellects to achieve even modest proficiency. It behooveth the future expert to remember this; and unless, from greater study, concentration and experience, we know the especial branch of medicine under which the case or cases in question come, say, I am not qualified to pass as an expert on this case or class of cases.

Men, who in private practice would unhesitatingly refer a case to some member of the profession who had given more time and study to certain branches of medicine than he had, will, without a moment's hesitation, get on the witness stand and belch forth most noisome opinions, sufficient to make the veriest tyro blush. Therefore the judges in courts of last resort have come to consider expert testimony the lowest order of evidence, instead of the highest and most reliable. They will be oblivious of the finesse necessary to expose malingering; ignorant of the fact that what is compatible with perfect sanity in a member of the humbler and lowlier, and possibly lowest walks of life, may be proof positive of insanity in a member of the higher walks and circles of society; blinded by a symptom complex, they may pronounce a person insane in whom the cremaster reflex may or may not be present; they will be deaf to the statement that a trauma may produce an organic or functional disease; ignorant of the difference between degeneracy and depravity; unable to differentiate the logical reasoning of the scholarly metaphysician from the unsystematized and fantastic delusions of the monomaniac; incapable of conceiving the fact that a man may, from education and association, consider as a truth the most exquisite absurdity, and because he, the prisoner, states that hell may have blocks of ice floating in it, is necessarily a hopeless dement, the witness believing as true propositions equally absurd.

Let us have men as experts. Men true to their convictions and conscious of their qualifications or deficiencies, and not assuming virtues they possess not.

The following selections from the evidence given by experts in several recent trials of murderers, for lunacy, are sufficient commentary: A prisoner charged with murder observes the strictest silence for many months, he is unwashed and unkempt; after every avenue of escape is closed, he having been convicted and sentenced, speech suddenly returns, and he tells the wise Æsculapians how he fooled them, when forthwith the aforesaid wise Æsculapians immediately pronounce him crazier than ever.

Another, who from his pronounced predilections for maidens not out of their teens, and who possesses that lack of perfect symmetry of physiognomy so common to the sons of men, is a degenerate, and consequently a paranoiac.

Still another, because he, too, was silent, and defecated on the floor of his cell (but never in the seat of his pants), was suffering from acute dementia.

From the foregoing remarks, I would not have any possible reader infer that I would for a moment cast one slur, nor would I stigmatize any gentleman witness, but rather that I would inculcate personal courage and integrity, and for those of us who may in the future be called upon to testify, do so to the best of our ability, not forgetting "that a fool doth think himself wise, while the wise man knoweth himself to be a fool."

Hugh Hagan, M.D.

